# Elemental stoichiometry and compositions of weevil larvae and two acorn hosts under natural phosphorus variation

**DOI:** 10.1038/srep45810

**Published:** 2017-04-05

**Authors:** Huawei Ji, Baoming Du, Chunjiang Liu

**Affiliations:** 1 School of Agriculture and Biology and Research Centre for Low-Carbon Agriculture, Shanghai Jiao Tong University, China; 2 Shanghai Urban Forest Research Station, State Forestry Administration, China; 3 Key Laboratory of Urban Agriculture (South), Ministry of Agriculture, China

## Abstract

To understand how different trophic organisms in a parasite food chain adapt to the differences in soil nutrient conditions, we investigated stoichiometric variation and homeostasis of multiple elements in two acorn trees, *Quercus variabilis* and *Quercus acutissima,* and their parasite weevil larvae (*Curculio davidi Fairmaire*) at phosphorus (P)-deficient and P-rich sites in subtropical China where P-rich ores are scattered among dominant P-deficient soils. Results showed that elemental stoichiometry and compositions of both acorns and weevil larvae differed significantly between P-deficient and P-rich sites (*p* < 0.05), with the largest contribution of acorn and weevil larva P in distinguishing the stoichiometric compositions between the two site types. The two acorn species were statistically separated by their acorn elemental stoichiometry and compositions (*p* < 0.05), but no difference was observed on weevil larvae between the two acorn species. P was one of the few elements that were non strict homeostasis in both acorns and weevil larvae. These findings highlight the importance of both environmental influence in elemental stoichiometry and composition and physiological regulations of nutritional needs in organisms and provide possible stoichiometric responses of both plants and animals to P loading, a worldwide issue from excess release of P into the environment.

The supplies of some key elements such as P (phosphorus) vary considerably in the natural environment, causing interspecific and intraspecific variations in elemental concentrations and ratios of organisms at different trophic levels^1^,^2^,^3^,^4^,^5^,^6^,^7^. For example, P rich soils have lower C:P and nitrogen (N):P ratios in plants^8^, higher P composition in plants^9^,^10^ and higher P and RNA content and population density of herbivore (*Sabinia setosa*)^11^. The higher soil inorganic nutrients (N, P, potassium (K), calcium (Ca), magnesium (Mg))^12^ on P rich sites may also lead to reduced locust outbreaks that occur more often on N-deficient soils^13^. Long-term adaptation to nutritional differences and resultant differentiation in stoichiometric composition helps ecotype formation^14^,^15^.

On the other hand, species are also capable of regulating elemental concentrations and ratios to maintain relatively constant stoichiometric composition with varying elemental supplies^5^,^7^,^16^,^17^,^18^,^19^. The degree of homeostatic regulation varies greatly among different stoichiometric traits^20^, trophic types^7^, and species^21^,^22^,^23^. Most homeostatic regulation studies, however, are concerned on individual species^9^,^20^,^23^; few have examined the responses of different trophic organisms in a food chain to varying element availability in the environment.

In subtropical China, P-rich sites developed on natural P-rich ores^20^,^24^ are surrounded by dominant P-deficient sites, and contain high contents of soil P and associated elements (e.g. Ca, Mg, Fe and aluminum (Al))^12^,^25^, providing an opportunity to study the stoichiometric plasticity and homeostatic regulation of organisms under different soil P availability. Research has indicated that plant species under these contrasting P conditions often develop ecotypes of distinct stoichiometric traits in P concentration and C:P ratios^14^,^15^,^20^,^26^,^27^,^28^. However, the understandings on ecological stoichiometry of geologic P variation require the examination of organisms at different trophic levels and on all life elements that are typically categorized by their biological functions, such as structure (C, N, P, S, Mg and Ca), electrochemistry (Na, K, P and Mg), mechanics (Ca, Mg and P), and catalysis (Fe, Cu, Zn and Mn)^4^,^29^.

In this study, we examined the stoichiometric elemental compositions of two acorn species, *Quercus variabilis* and *Quercus acutissima*, and associated parasite weevil larvae (Curculio davidi Fairmaire) that complete their last developmental stage in a single host acorn, in Central Yunnan Plateau, subtropical China. Within its natural distribution, weevil larva is also associated with *Castanea mollissima, Castanea henryi* and *Castanea seguinii*^3^,^30^,^31^. We measured stoichiometric variation and homeostasis of 13 elements (C, N, P, S, K, Ca, Mg, Fe, Mn, Zn, Cu, Al and Na) in soils, acorns and weevil larvae. We hypothesized that (i) both acorns and weevil larvae would have significant interspecific and intraspecific variations in elemental stoichiometry and compositions and therefore nutrient-based stoichiometric traits through long-term adaptation to natural soil P variation, (ii) P would be the dominant element influencing the intraspecific variations of the acorn and weevil larva stoichiometric composition between the two site types of contrasting P, (iii) the degree of stoichiometric homeostasis would differ among different stoichiometric traits, between two acorn species, and between acorns and weevil larvae due to different nutritional demands and biological functions by elements, species, and trophic levels.

## Results

### Soil stoichiometry

**Figure 1:**
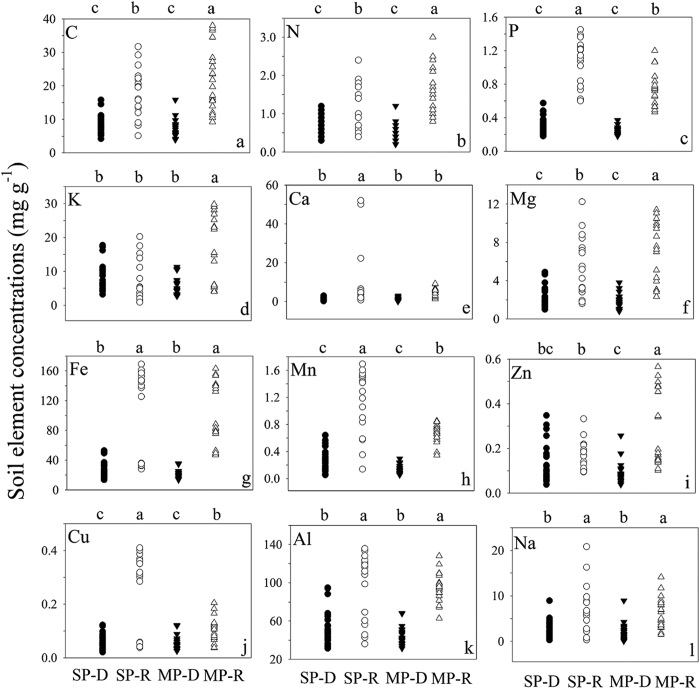
Soil element concentrations in *Q. variabilis* at P-deficient sites (SP-D) and P-rich sites (SP-R) and in *Q. acutissima* at P-deficient sites (MP-D) and P-rich sites (MP-R) in central Yunnan Plateau, Southwest China. Each site and acorn species combination had soil element concentrations at 0–10 cm, 10–20 cm and 20–30 cm depths. Elements with different letters differ significantly (*p* < 0.05) among the four groups of acorn species by site combinations, SP-D, SP-R, MP-D and MP-R.

In both *Q. variabilis* and *Q. acutissima* stands, the soil element concentrations at P-rich sites were higher than those at P-deficient sites (*p* < 0.05), with the exception of K and Zn in *Q. variabilis* stands and Ca in *Q. acutissima* stands that did not significantly differ between the two site types (Fig. 1).

### Acorn stoichiometry

**Figure 2:**
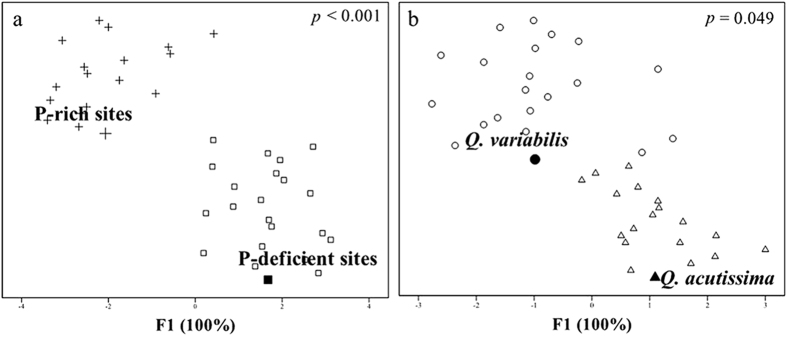
Plots of single discriminant function by discriminant functional analysis on acorn C, N, P, S, K, Ca, Mg, Fe, Mn, Zn, Cu, Al and Na concentrations in separations between P-deficient sites (squares) and P-rich sites (plus) (**a**) and between *Q. variabilis* (circles) and *Q. acutissima* species (triangles) (**b**).

**Figure 3:**
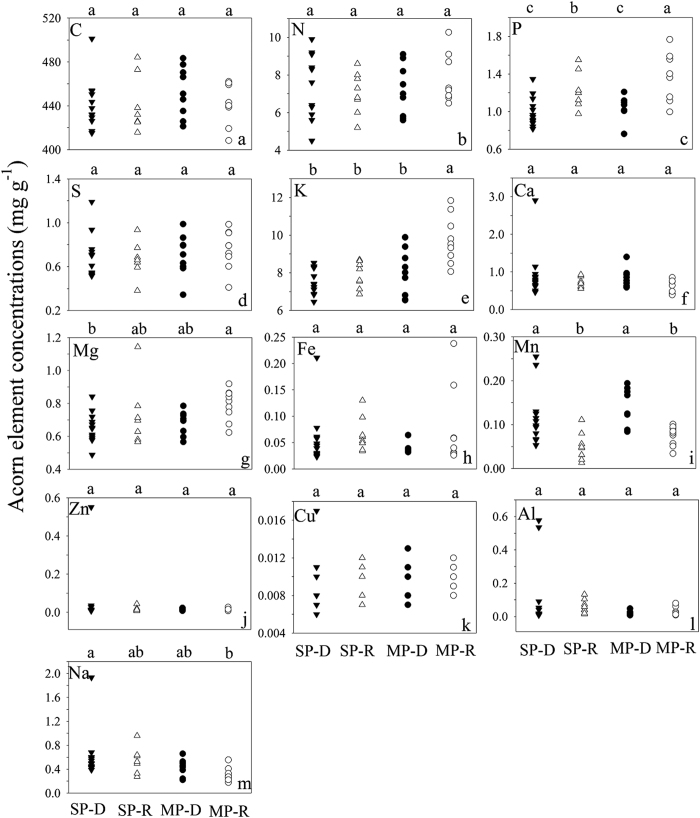
Acorn element concentrations in *Q. variabilis* at P-deficient sites (SP-D) and P-rich sites (SP-R) and in *Q. acutissima* at P-deficient sites (MP-D) and P-rich sites (MP-R) in central Yunnan Plateau, Southwest China. Elements with different letters differ significantly (*p* < 0.05) among the four groups of acorn species by site combinations, SP-D, SP-R, MP-D and MP-R.

**Figure 4:**
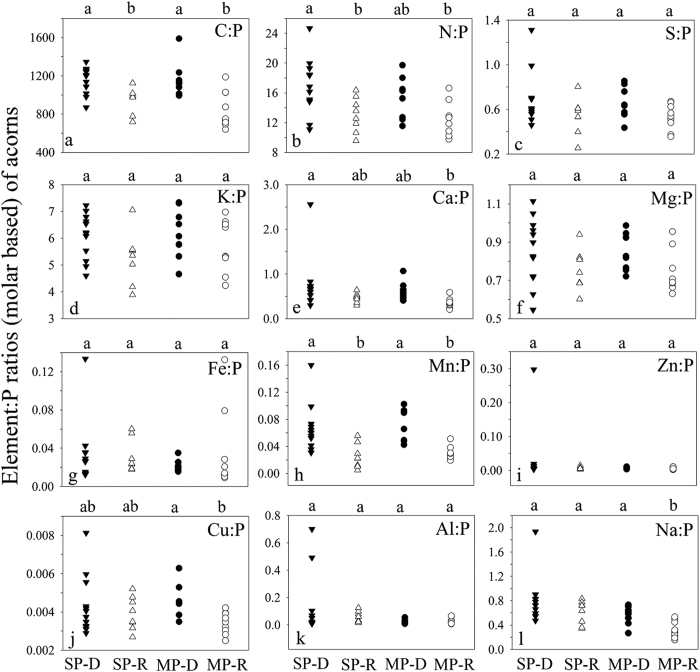
Acorn element:P ratios (molar) in *Q. variabilis* at P-deficient sites (SP-D) and P-rich sites (SP-R) and in *Q. acutissima* at P-deficient sites (MP-D) and P-rich sites (MP-R) in central Yunnan Plateau, Southwest China. Elements with different letters differ significantly (*p* < 0.05) among the four groups of acorn species by site combinations, SP-D, SP-R, MP-D and MP-R.

The two acorn species were statistically separated on their acorn elemental composition between P-rich and P-deficient sites based on the DFA analysis (F1 explained 100% of the variance, *p* < 0.001) (Fig. 2a, Table 1). The two site types were also discriminated by acorn P, K, Mg and Mn concentrations and the ratios of P with most elements (except Fe, Zn and Al) (*p* < 0.05) (Figs 3 and 4, Table 2).

*Q. variabilis* species was statistically separated on acorn elemental composition from *Q. acutissima* along F1 (*p* = 0.049) (Fig. 2b). Acorn K and Na concentrations and Na:P ratios also differed between *Q. variabilis* and *Q. acutissima* (Figs 3 and 4, Table 2).

### Weevil larva stoichiometry

**Figure 5:**
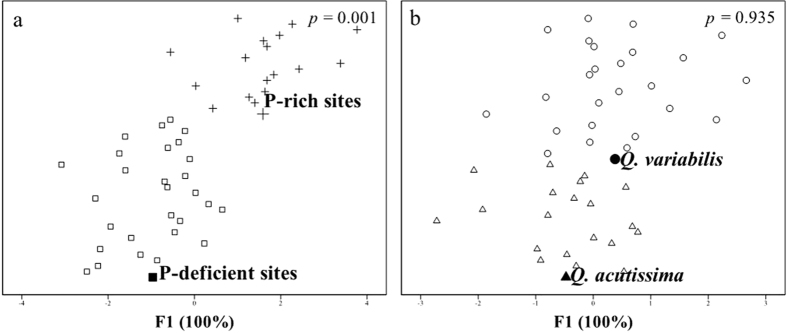
Plots of single discriminant function by discriminant functional analysis on weevil larva C, N, P, S, K, Ca, Mg, Fe, Mn, Zn, Cu, Al and Na concentrations in separations between P-deficient sites (squares) and P-rich sites (plus) (**a**) and between *Q. variabilis* (circles) and *Q. acutissima* species (triangles) (**b**).

**Figure 6:**
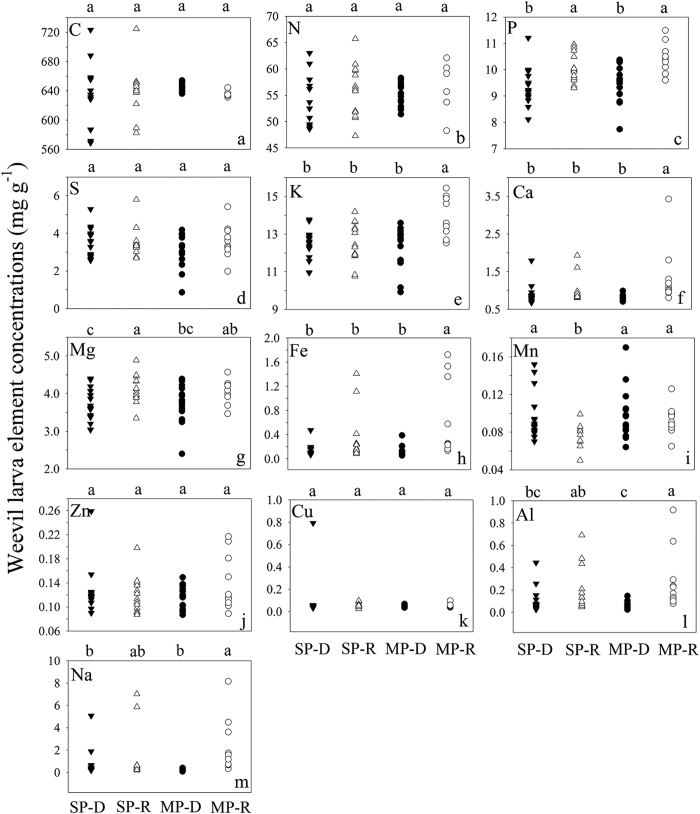
Weevil larva element concentrations in *Q. variabilis* at P-deficient sites (SP-D) and P-rich sites (SP-R) and in *Q. acutissima* at P-deficient sites (MP-D) and P-rich sites (MP-R) in central Yunnan Plateau, Southwest China. Elements with different letters differ significantly (*p* < 0.05) among the four groups of acorn species by site combinations, SP-D, SP-R, MP-D and MP-R.

**Figure 7:**
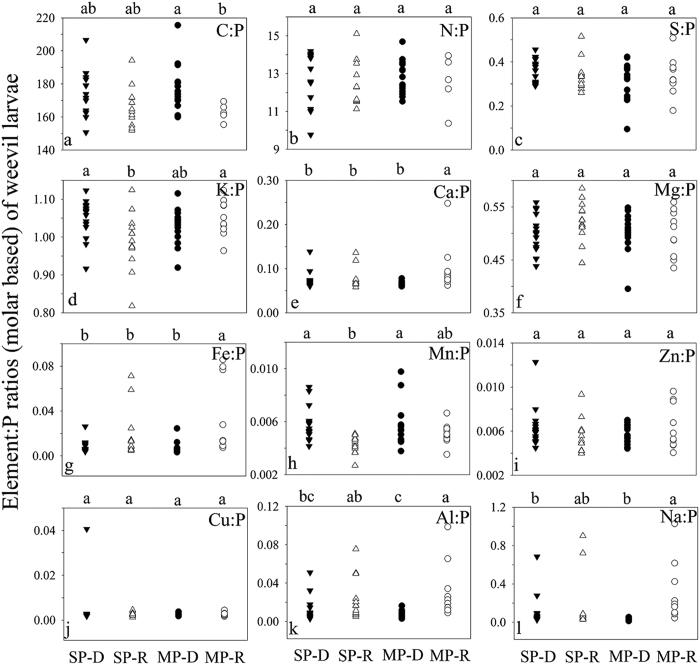
Weevil larva element:P ratios (molar) in *Q. variabilis* at P-deficient sites (SP-D) and P-rich sites (SP-R) and in *Q. acutissima* at P-deficient sites (MP-D) and P-rich sites (MP-R) in central Yunnan Plateau, Southwest China. Elements with different letters differ significantly (*p* < 0.05) among the four groups of acorn species by site combinations, SP-D, SP-R, MP-D and MP-R.

The weevil larvae at the P-deficient sites were statistically separated from those at P-rich sites on larva elemental composition along F1 (*p* = 0.001) (Fig. 5a), as well as on their P, Ca, Mg, Fe, Mn, Al and Na concentrations and C:P, Fe:P, Mn:P, Al:P and Na:P ratios (Figs 6 and 7, Table 3).

The weevil larvae were not statistically separated between *Q. variabilis* and *Q. acutissima* on larva elemental composition based on DFA analysis (*p* = 0.935) (Fig. 5b). Weevil larvae were not discriminated by any of their elements and element:P ratios between the two host acorn species (Figs 6 and 7, Table 3).

### Homeostasis of elements in acorns and weevil larvae

Most elements and element:P ratios were strictly homeostatic in both acorns and weevil larvae, with exceptions in acorn P (homeostatic), acorn Cu:P (homeostatic), weevil larva P (weakly homeostatic), weevil larva Mn (homeostatic), weevil larva Na (homeostatic), weevil larva C:P (weakly plastic), weevil larva K:P (homeostatic) and weevil larva Mn:P (homeostatic) in *Q. variabilis*, and acorn P (homeostatic), acorn K (homeostatic), acorn Ca (weakly homeostatic), acorn Mn (weakly homeostatic), acorn Ca:P (weakly homeostatic), weevil larva P (homeostatic), weevil larva Ca (plastic), weevil larva Fe (plastic), and weevil larva Ca:P (weakly homeostatic) in *Q. acutissima* (Tables 4 and 5, Supplementary Figs S1 and S2).

## Discussion

Our hypothesis on nutrient-based stoichiometric traits by natural soil P variation is accepted. As with the study by Zhou, *et al*.^20^ on acorn leaf stoichiometric traits, some elemental concentrations and ratios of both acorn seeds and weevil larvae differed significantly between P-rich sites and P-deficient sites, likely due to the differences in soil P, Ca, Mg, Fe, Mn and Al between the two site types^12^,^20^ (Figs 3, 4 and 6 and 7, Supplementary Tables S1, S2, S3 and S4). This suggests stoichiometric plasticity of both plants and their parasite in response to elemental variability in the environment^4^, as well as a strong influence of soil elements on different trophic organisms along a food chain. Compared to nutrient addition experiments that often examine short-term acclimation to the environment^32^, our study revealed long-term adaptations to elemental variations between P-rich sites and P-deficient sites^20^,^28^,^32^, possibly through phenotypic plasticity and genetic differentiation^33^, the key mechanisms for ecotype formation^14^,^15^.

According to the growth rate hypothesis^34^,^35^,^36^, the acorns and weevil larvae at the P-rich sites would have higher gowth rates and productivity due to their higher P concentrations and lower C:P ratios (Figs 3, 4 and 6 and 7). Similarly, Schade *et al*.^11^ found higher plant C:P ratio and lower P and RNA content and population dynamics of a herbivore (*Sabinia setosa*) on P-deficient soil. The long-term influences of P availability on algae^37^, terrestrial plants^38^ and herbivorous animals^6^ suggest that the geologic P variation between the two site types may change not only elmental stoichiometry but also productivity and population dynamics or even genetic adaptation of acorn trees and weevil larvae along a plant-parasite food chain.

Our expectations on the dominant influence of P on intraspecific variations and stoichiometric homeostasis are generally true. Among the elements examined, acorn P and weevil larva P had the largest contribution in distinguishing acorn and weevil larva stoichiometric composition, respectively, between the P-deficient and P-rich sites (Table 1), although other elements and elemental ratios such as Ca, Mg, Fe, Mn, Al, and Na also significantly distinguished the stoichiometric composition between the two site types of contrasting P (Figs 2a and 5a, Tables 1, 2 and 3). The strong stoichiometric plasticity of acorn plants and weevil larvae in their responses to P variation was also supported by the results of homeostasis analysis which indicated that P was one of the few elements that were non strict homeostasis in both acorns and weevil larvae (Table 4) and more likely varied with P availability in the environment.

Compared to the intraspecific variations of stoichiometric composition in acorns and weevil larvae that were mainly influenced by P, the interspecific variations between the two acorn tree species were dominated by Na (Fig. 2b, Tables 1 and 2), consistent to the findings that Na responds most to interspecific variations in higher plants^39^. In our study, soil Na content did not differ between the locations of two acorn tree species and there was no significant interaction between site types and acorn species on acorn Na (Supplementary Table S5). Thus, the interspecific variation of acorn Na between *Q. variabilis* and *Q. acutissima* was due to their different needs in metabolism and physiology^5^ and likely the strict homeostatic regulation of acorn Na (Table 4)^7^,^18^,^19^. The differential needs of Na between two acorn species may be ecologically significant in minimizing interspecific competition and increasing species diversity^40^.

This may be also true for weevil larvae that maintained elemental composition in different hosting environments of *Q. variabilis* and *Q. acutissima* through homeostatic regulation (Fig. 5b, Table 3). Across two *Q. variabilis* and *Q. acutissima* stands and two site types and comparatively, macro-elements were more strictly regulated in weevil larvae than in acorns relative to some essential microelements such as Fe, Cu and Na (Supplementary Table S6), consistent to the findings by others that herbivorous consumers are generally lower in stoichiometric flexibility than plants^41^,^42^,^43^, and that essential microelements are weakly regulated and have high somatic variation in many invertebrates^18^,^44^,^45^,^46^,^47^. The different stoichiometric flexibility between macroelements and microelements in weevil larvae across two *Q. variabilis* and *Q. acutissima* stands and two site types (Supplementary Table S6) indicates that homeostatic regulation varies with element types (Table 4), probably due to their different stoichiometric composition and physiological functions^7^,^18^.

In our study, the degree of homeostatic regulation varied with stoichiometric traits, species, and trophic levels and ranged from “strict homeostasis” to “plastic” (Tables 4 and 5, Supplementary Figs S1 and S2). A strict homeostasis is important for meeting nutritional demands and maintaining biological functions^32^, as shown on most stoichiometric traits of both acorns and weevil larvae (Tables 4 and 5). Non-homeostatic responses such as acclimation or adaptation may be favored if strict homeostasis does not allow organism to survive for long-term stressful environment^32^. To large extent, physiological mechanisms (such as homeostasis) are strongly linked to genetic mechanism and evolution of nutritional demands for animal growth^6^,^48^,^49^,^50^. Therefore, the non-strict homeostasis of some elements and element ratios, especially P, in this study (Tables 4 and 5, Supplementary Figs S1 and S2) may give organisms capacity to adapt to nutrient variability and increase species fitness^51^.

In summary, the elemental stoichiometric tratits of both acorns and the parasite weevil larvae were strongly influenced by natural soil P variation, as well as by homeostatic regulation, resulting in considerable intraspecific and interspecific variations. Among all the elements examined, P in acorns and weevil larvae was dominant in distinguishing the stoichiometric composition of acorns and weevil larvae between the P-deficient and P-rich sites. The degree of homeostatic regulation varied with stoichiometric traits, species, and trophic levels and ranged from “strict homeostasis” to “plastic”. The strict homeostatic regulation would be important for maintaining species-specific elemental composition, while the non-strict homeostasis of some elements and element ratios, especially acorn P and weevil larva P, may be critical for both acorn plants and the parasite to adapt to the environment of varying element (P) concentrations. These findings highlight the importance of both environmental influence in elemental stoichiometry and composition and physiological regulations of nutritional needs in organisms. The results will also help understand possible stoichiometric responses of both plants and animals to P loading, a worldwide issue from excess release of P into the environment^24^,^52^.

## Methods

### Study area and materials

**Figure 8:**
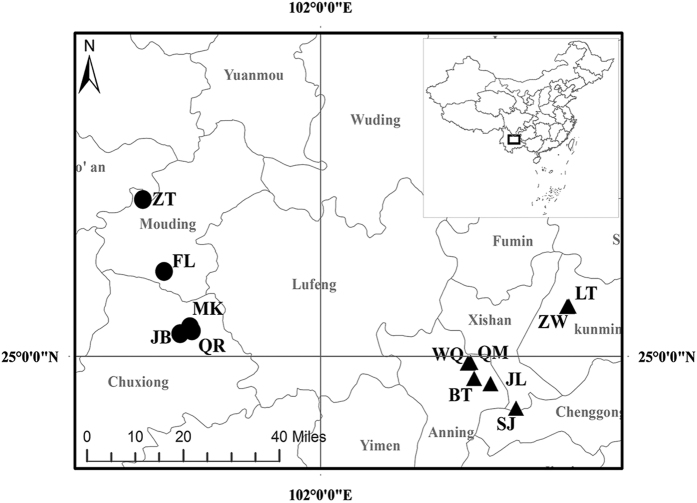
The locations of sampling sites including 8 *Quercus variabilis* sites (LT, ZW, SJ, WQ, FL, ZT, QR and JB) and 6 *Quercus acutissima* sites (QM, BT, JL, QR, JB and MK) in Yunnan province. Circles represent for P-deficient sites and triangles for P-rich sites. The map was drawn by the author of Huawei Ji with ArcGIS 10.2 (ESRI): www.esri.com/software/arcgis/.

The study area is located in central Yunnan Plateau, subtropical China where P-rich and P-deficient sites are available for studying elemental stoichiometry of acorns and parasite weevil larvae under different environmental conditions^20^,^24^. P-rich sites (developed on P-rich phosphate rocks) are located at Kunming City where P-rich ores are distributed and P-deficient sites (developed on non-phosphate rocks) are located at Chuxiong City (Fig. 8 and Supplementary Table S7) (see typical chemical compositions of phosphate rocks on P-rich sites^25^ and non-phosphate rocks on P-deficient sites^53^ in Supplementary Table S8). Climate is similar between the two site types, with a mean annual temperature of 15.4 °C and mean annual precipitation of 936.5 mm. In the study area, *Q. variabilis* and *Q. acutissima* occur in pure or mixed stands of each other or with other tree species. The two acorn trees are the common hosts of parasite *C. davidi* larvae in the study area.

### Preparation of soil, acorn and weevil larva samples

Four *Q. variabilis* and three *Q. acutissima* stands were selected for each of the P-rich and P-deficient site types (Fig. 8). Three 20 m × 20 m plots were established within each stand. Soil was sampled to three depths, 0 to 10 cm, 10 to 20 cm, and 20 to 30 cm, and the samples of the same depth from 5 locations in a plot were mixed to form a composite sample. The soil samples were air-dried and sieved through a 60 mesh sieve (0.25 mm diameter) for chemical analysis.

About 300 acorns were collected on the ground of each selected plots and transported to the laboratory during the period of peak falling in October of 2014. In each plot, a minimum of 5αcorns that were healthy and well-developed and not infested by weevil were combined to make a composite acorn sample for chemical analysis, and the remaining of acorns were stored at the room temperature for larva collection. The composite acorn samples for chemical analysis were scarified with a knife to remove pericarp to obtain seeds, which were then dried at 65 °C, triturated with a blade mill to obtain a fine powder, and sieved through a 60 mesh sieve for chemical analysis.

Additional two 20 m × 20 m plots were added within each stand if inadequate larvae parasitizing was noticed in the first three plots. Larvae were immediately collected when they first crawled out of the acorns, frozen in liquid N_2_, and preserved in a freezer at −80 °C for chemical analysis. In total, 38 acorn samples and 55 weevil larva samples were obtained for chemical analysis. The sampled larvae ranged from 6 to 10 mm in body length and from 30 to 80 mg in body mass (dry weight).

### Chemical analysis

Soil, acorn and weevil larva samples were then digested with trace metal-grade nitric acid and diluted with distilled water. Total C and N concentrations were analyzed with an elemental analysis-stable isotope ratio mass spectrometer (EAI) (Vario ELIII; Elementar, Germany), while total P, S, K, Ca, Mg, Fe, Mn, Zn, Cu, Al and Na were determined with a plasma optical emission spectrometer (ICP-OES) (Iris Advantage 1000; Thermo Jarrell Ash, Franklin, MA) at the Instrumental Analysis Centre of Shanghai Jiao Tong University. For EAI analysis, the larvae were dried at 50 °C until constant weight and then ≥7 weevil larvae were grinding with a homogenizer at 5000 r min^−1^ to make a composite weevil larva sample per plot. For ICP-OES analysis, about 20 dried larvae were combined directly (without grinding) to make a composite weevil larva sample per plot. The detection limits and standards for all elements were provided in Supplementary Table S9. The element concentrations of all samples were in milligrams per gram (mg g^−1^) of dry weight.

### Homeostasis calculation

The degree of stoichiometric homeostasis was expressed with the homeostasis coefficient H described by ref. 7


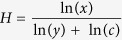


In acorns x is the element concentration or ratio in soils and y is the corresponding element concentration or ratio in acorns, whereas in weevil larvae x is the element concentration or ratio in acorns and y is the corresponding element concentration or ratio in weevil larvae. 1/H is the slope of the regression between log_e_(y) and log_e_(x) and indicates the regression relationships between y and x^2^. The degree of homeostasis of an element or the element:P ratio is ‘strictly homeostatic’ when regression *p* ≥ 0.1, ‘homeostatic’ when 0 < 1/H < 0.25, ‘weakly homeostatic’ when 0.25 < 1/H < 0.5, ‘weakly plastic’ when 0.5 < 1/H < 0.75, and ‘plastic’ when 1/H > 0.75 and regression *p* < 0.1^2^.

### Statistical analyses

First, analysis of variance (ANOVA) was used with R version 3.2.0 (The R Foundation for Statistical Computing, 2015) to examine the differences of soil element concentrations between two site types and between two acorn species (each site and acorn species (stand) was measured for soil element concentrations at 0–10 cm, 10–20 cm and 20–30 cm depths), and the differences of element concentrations and element:P ratios of acorns and weevil larvae between site types and between acorn species. Scatterplots (Jitter diagram) also were depicted by SigmaPlot 10.0 (Systat software, Inc., 2006) to show the element concentrations or element:P ratios of soils, acorns and weevil larvae. Second, discriminant functional analysis (DFA) was applied with SPSS 18.0 (SPSS Inc., USA) using acorn C, N, P, S, K, Ca, Mg, Fe, Mn, Zn, Cu, Al and Na concentrations and element: P ratios from 38 plots or weevil larva C, N, P, S, K, Ca, Mg, Fe, Mn, Zn, Cu, Al and Na concentrations and element: P ratios from 45 plots (10 samples were inadequate for C and N determination) to assess the levels of considerable interspecific and intraspecific variations. Third, the stoichiometric homeostasis of both acorns and weevil larvae was calculated through regression analysis using SigmaPlot 10.0 (Systat software, Inc., 2006) to access if stoichiometric homeostasis was related to soil elemental differences. Map was drawn with ArcGIS 10.2 (ESRI): www.esri.com/software/arcgis/.

## Additional Information

**How to cite this article**: Ji, H. *et al*. Elemental stoichiometry and compositions of weevil larvae and two acorn hosts under natural phosphorus variation. *Sci. Rep.* **7**, 45810; doi: 10.1038/srep45810 (2017).

**Publisher's note:** Springer Nature remains neutral with regard to jurisdictional claims in published maps and institutional affiliations.

## Supplementary Material

Supplementary Figures and Tables

## Figures and Tables

**Table 1:** Standardized coefficients of F1 from discriminant functional analysis in separation of acorn and weevil larva C, N, P, S, K, Ca, Mg, Fe, Mn, Zn, Cu, Al and Na concentrations.

Elements	Acorns		Weevil larvae	
	Separation between P-deficient sites and P-rich sites	Separation between *Q. variabilis* and *Q. acutissima* species	Separation between P-deficient sites and P-rich sites	Separation between *Q. variabilis* and *Q. acutissima* species
C	0.317	0.343	−0.237	−0.286
N	0.517	−0.230	0.222	0.057
P	−1.250	0.041	0.988	−0.358
S	0.286	0.218	−0.180	0.715
K	−0.154	0.502	−0.506	−0.435
Ca	0.238	−0.065	0.342	−0.730
Mg	0.299	0.566	0.006	0.755
Fe	−0.801	0.107	0.338	−0.087
Mn	0.796	0.875	−0.548	−0.260
Zn	0.955	−0.559	−0.414	−0.085
Cu	−0.323	−0.018	−0.247	0.434
Al	−1.079	0.523	0.159	0.719
Na	0.713	−1.307	0.383	0.021
% Variance explained	100%	100%	100%	100%

**Table 2:** Results (Wilks’ Lambda and *p*-value) of discriminant functional analysis on acorn C, N, P, S, K, Ca, Mg, Fe, Mn, Zn, Cu, Al and Na concentrations and C:P, N:P, S:P, K:P, Ca:P, Mg:P, Fe:P, Mn:P, Zn:P, Cu:P, Al:P and Na:P molar ratios in separations between P-deficient sites and P-rich sites and between *Q. variabilis* and *Q. acutissima* species.

Independent variables	Dependent variables of FDA analysis			
	Separation between P-deficient sites and P-rich sites		Separation between *Q. variabilis* and *Q. acutissima* species	
	Wilks’ Lambda	*p*-level	Wilks’ Lambda	*p*-level
C	0.98	0.415	0.97	0.338
N	1.00	0.940	1.00	0.797
P	0.63	**0.000**	0.93	0.103
S	1.00	0.806	0.98	0.380
K	0.80	**0.005**	0.78	**0.003**
Ca	0.92	0.093	0.97	0.343
Mg	0.85	**0.016**	0.95	0.164
Fe	0.95	0.177	1.00	0.852
Mn	0.67	**0.000**	0.98	0.454
Zn	0.98	0.391	0.98	0.347
Cu	1.00	0.738	0.97	0.330
Al	0.98	0.428	0.91	0.075
Na	0.96	0.212	0.86	**0.020**
C:P	0.61	**0.000**	0.96	0.259
N:P	0.73	**0.001**	0.96	0.236
S:P	0.87	**0.026**	0.99	0.569
K:P	0.89	**0.041**	0.99	0.574
Ca:P	0.85	**0.015**	0.96	0.214
Mg:P	0.86	**0.020**	0.99	0.654
Fe:P	0.99	0.578	1.00	0.684
Mn:P	0.56	**0.000**	1.00	0.828
Zn:P	0.97	0.338	0.97	0.338
Cu:P	0.86	**0.021**	1.00	0.882
Al:P	0.97	0.338	0.91	0.075
Na:P	0.83	**0.010**	0.78	**0.003**

**Table 3:** Results (Wilks’ Lambda and *p*-value) of discriminant functional analysis on weevil larva C, N, P, S, K, Ca, Mg, Fe, Mn, Zn, Cu, Al and Na concentrations and C:P, N:P, S:P, K:P, Ca:P, Mg:P, Fe:P, Mn:P, Zn:P, Cu:P, Al:P and Na:P molar ratios in separations between P-deficient sites and P-rich sites and between *Q. variabilis* and *Q. acutissima* species.

Independent variables	Dependent variables of FDA analysis			
	Separation between P-deficient sites and P-rich sites		Separation between *Q. variabilis* and *Q. acutissima* species	
	Wilks’ Lambda	*p*-level	Wilks’ Lambda	*p*-level
C	1.00	0.859	0.99	0.455
N	0.93	0.082	1.00	0.781
P	0.84	**0.007**	1.00	0.969
S	0.96	0.205	0.98	0.302
K	0.95	0.146	1.00	0.652
Ca	0.74	**0.000**	1.00	0.691
Mg	0.85	**0.008**	1.00	0.915
Fe	0.77	**0.001**	1.00	0.792
Mn	0.88	**0.022**	0.96	0.190
Zn	1.00	0.866	1.00	0.794
Cu	0.99	0.511	0.99	0.434
Al	0.78	**0.001**	0.97	0.264
Na	0.73	**0.000**	1.00	0.993
C:P	0.86	**0.011**	0.99	0.586
N:P	0.99	0.527	1.00	0.784
S:P	0.99	0.505	0.97	0.233
K:P	0.96	0.170	0.99	0.577
Ca:P	0.94	0.112	0.99	0.633
Mg:P	0.93	0.079	1.00	0.907
Fe:P	0.83	**0.004**	1.00	0.810
Mn:P	0.80	**0.002**	0.96	0.180
Zn:P	0.98	0.393	1.00	0.857
Cu:P	0.99	0.459	0.99	0.436
Al:P	0.79	**0.002**	0.97	0.235
Na:P	0.77	**0.001**	1.00	0.932

**Table 4:** Homeostasis (1/*H*) of acorn elements in *Q. variabilis* (ASP) and *Q. acutissima* (AMP), and homeostasis (1/*H*) of weevil larva elements in *Q. variabilis* (WSP) and *Q. acutissima* (WMP) across all variable element sites in Central Yunnan Plateau, southwest China.

Elements	ASP			WSP			AMP			WMP		
	1/H	SE	*p*	1/H	SE	*p*	1/H	SE	*p*	1/H	SE	*p*
C	—	—	—	0.21	0.274	0.457	—	—	—	0.071	0.056	0.234
N	0.104	0.137	0.459	0.020	0.106	0.850	0.004	0.065	0.949	0.053	0.109	0.637
P	**0.125**	**0.057**	**0.046**	**0.308**	**0.059**	**<0.001**	**0.217**	**0.084**	**0.024**	**0.163**	**0.075**	**0.046**
S	—	—	—	0.103	0.181	0.574	—	—	—	0.238	0.358	0.516
K	0.031	0.033	0.358	0.001	0.190	0.996	**0.100**	**0.054**	**0.091**	0.207	0.146	0.176
Ca	0.055	0.127	0.670	0.021	0.181	0.910	**0.264**	**0.079**	**0.006**	**0.773**	**0.224**	**0.004**
Mg	0.037	0.073	0.616	0.237	0.162	0.162	0.042	0.047	0.384	0.048	0.193	0.809
Fe	0.106	0.189	0.585	0.024	0.353	0.947	0.226	0.201	0.282	**0.948**	**0.420**	**0.039**
Mn	0.223	0.197	0.278	**0.188**	**0.045**	**<0.001**	**0.302**	**0.123**	**0.031**	0.107	0.128	0.416
Zn	0.329	0.533	0.547	0.002	0.063	0.971	0.252	0.150	0.119	0.211	0.175	0.246
Cu	0.061	0.080	0.459	0.442	0.592	0.464	0.055	0.118	0.651	0.439	0.342	0.219
Al	0.400	0.692	0.573	0.023	0.182	0.903	0.124	0.492	0.804	0.269	0.387	0.497
Na	0.114	0.094	0.249	**1.474**	**0.574**	**0.019**	0.121	0.165	0.476	0.455	0.867	0.607

**Table 5:** Homeostasis (1/*H*) of acorn element:P ratios in *Q. variabilis* (ASP) and *Q. acutissima* (AMP), and homeostasis (1/*H*) of weevil larva element:P ratios in *Q. variabilis* (WSP) and *Q. acutissima* (WMP) across all variable element sites in Central Yunnan Plateau, southwest China.

Element:P ratios	ASP			WSP			AMP			WMP		
	1/H	SE	*p*	1/H	SE	*p*	1/H	SE	*p*	1/H	SE	*p*
C:P	—	—	—	**0.566**	**0.133**	**<0.001**	—	—	—	0.114	0.084	0.205
N:P	0.111	0.148	0.466	0.079	0.101	0.449	0.144	0.196	0.476	0.164	0.096	0.118
S:P	—	—	—	0.002	0.128	0.988	—	—	—	0.511	0.377	0.195
K:P	0.041	0.036	0.278	**0.176**	**0.083**	**0.047**	0.100	0.061	0.128	0.021	0.076	0.788
Ca:P	0.308	0.198	0.145	0.106	0.132	0.433	**0.458**	**0.178**	**0.024**	**0.439**	**0.180**	**0.027**
Mg:P	0.110	0.080	0.190	0.013	0.101	0.898	0.015	0.071	0.835	0.017	0.120	0.891
Fe:P	0.361	0.403	0.386	0.228	0.310	0.471	0.303	0.450	0.514	0.526	0.388	0.195
Mn:P	0.121	0.381	0.756	**0.222**	**0.039**	**<0.001**	0.279	0.276	0.332	0.176	0.106	0.118
Zn:P	0.099	0.439	0.825	0.007	0.061	0.911	0.422	0.250	0.117	0.056	0.130	0.676
Cu:P	**0.242**	**0.107**	**0.042**	0.154	0.578	0.793	0.107	0.197	0.596	0.040	0.259	0.879
Al:P	0.268	0.776	0.736	0.048	0.179	0.793	0.953	0.635	0.159	0.075	0.349	0.832
Na:P	0.039	0.099	0.701	0.833	0.673	0.232	0.042	0.210	0.845	0.625	0.634	0.340
