# Supplementary material for: Elemental stoichiometry and compositions of weevil larvae and two acorn hosts under natural phosphorus variation
Source: Sci Rep. 2017 Apr 5;7:45810. doi: 10.1038/srep45810 (PMC5381121; doi:10.1038/srep45810)
Supplement: Supplementary Figures and Tables [file srep45810-s1.pdf]

Elemental stoichiometry and compositions of weevil larvae and two acorn hosts under natural phosphorus variation

Huawei Ji[1,2], Baoming Du[1,2], Chunjiang Liu[1,2,3*]

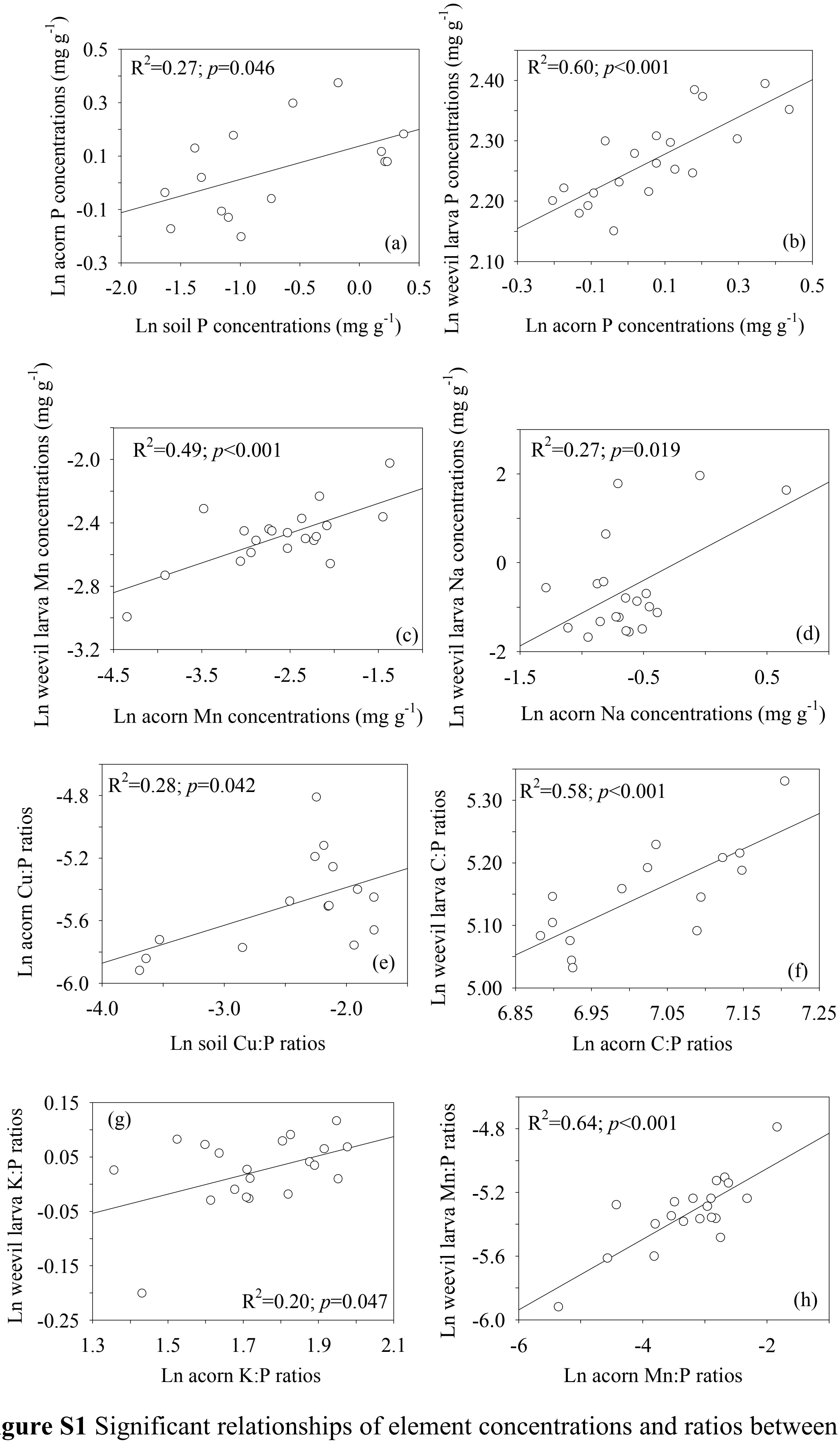

Figure S1 Significant relationships of element concentrations and ratios between soils and acorns or between acorns and weevil larvae in *Q. variabilis*.

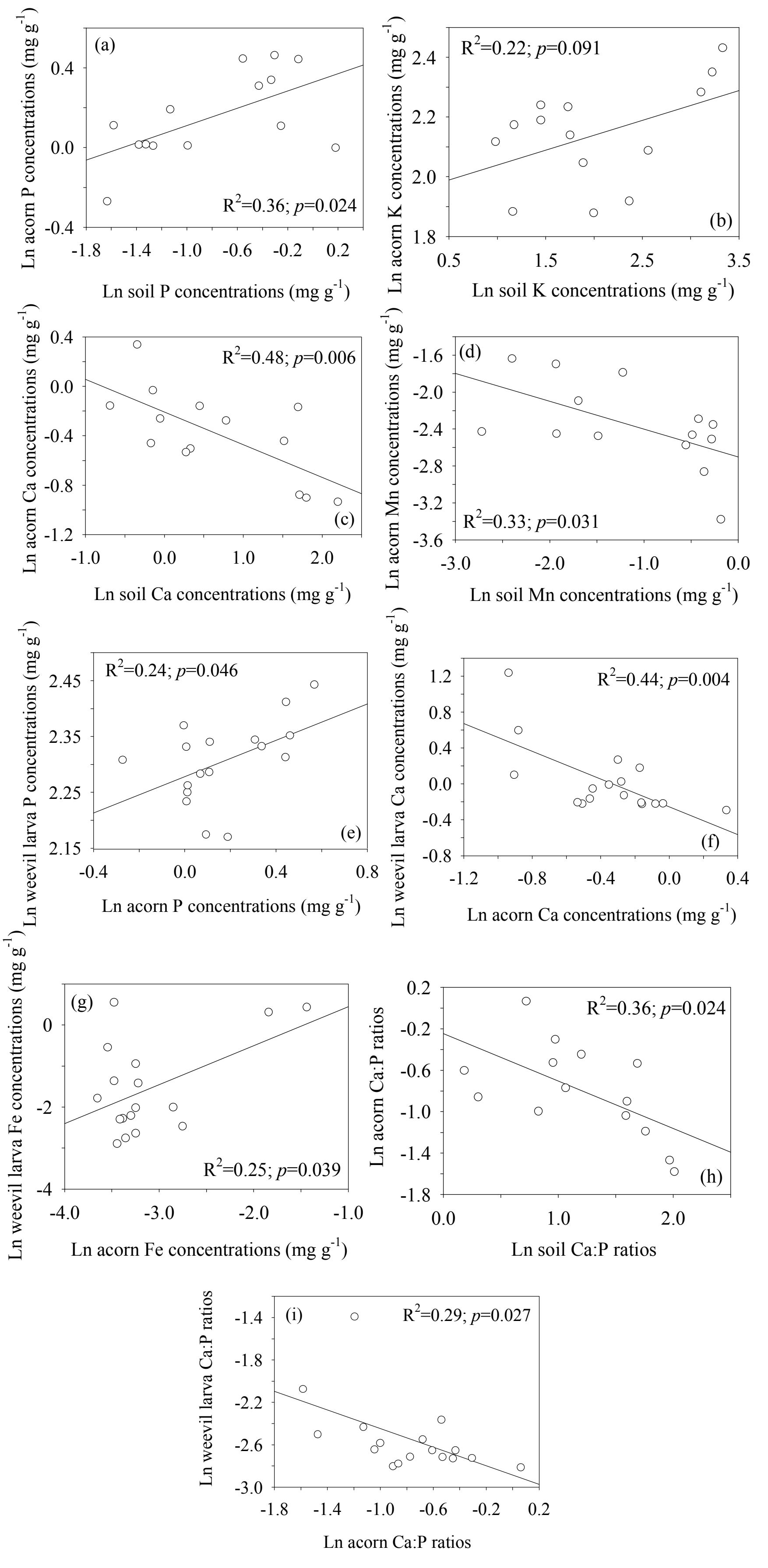
(a)
R2=0.36; p=0.024
Ln acorn P concentrations (mg g-1)
Ln soil P concentrations (mg g-1)
(b)
R2=0.22; p=0.091
Ln acorn K concentrations (mg g-1)
Ln soil K concentrations (mg g-1)
(c)
R2=0.48; p=0.006
Ln acorn Ca concentrations (mg g-1)
Ln soil Ca concentrations (mg g-1)
(d)
R2=0.33; p=0.031
Ln acorn Mn concentrations (mg g-1)
Ln soil Mn concentrations (mg g-1)
(e)
R2=0.24; p=0.046
Ln weevil larva P concentrations (mg g-1)
Ln acorn P concentrations (mg g-1)
(f)
R2=0.44; p=0.004
Ln weevil larva Ca concentrations (mg g-1)
Ln acorn Ca concentrations (mg g-1)
(g)
R2=0.25; p=0.039
Ln weevil larva Fe concentrations (mg g-1)
Ln acorn Fe concentrations (mg g-1)
(h)
R2=0.36; p=0.024
Ln acorn Ca:P ratios
Ln soil Ca:P ratios
(i)
R2=0.29; p=0.027
Ln weevil larva Ca:P ratios
Ln acorn Ca:P ratios

Figure S2 Significant relationships of element concentrations and ratios between soils and acorns or between acorns and weevil larvae in *Q. acutissima*.

Table S1 Acorn element concentrations (mg g^{-1}) of *Q. variabilis* and *Q. acutissima* at P-deficient and P-rich sites in central Yunnan Plateau, Southwest China. Means with different letters differ significantly ($p < 0.05$) among the four groups of acorn species by site combinations.

Acorns	*Q. variabilis*				*Q. acutissima*			
	P-deficient sites		P-rich sites		P-deficient sites		P-rich sites	
	Mean	SE	Mean	SE	Mean	SE	Mean	SE
C	440.98a	6.64	438.75a	9.15	452.99a	7.56	441.28a	6.14
N	7.53a	0.50	7.05a	0.39	7.18a	0.45	7.74a	0.43
P	1.00c	0.05	1.21b	0.07	1.03c	0.04	1.39a	0.09
S	0.69a	0.06	0.66a	0.06	0.70a	0.06	0.76a	0.06
K	7.54b	0.20	7.88b	0.25	8.00b	0.41	9.76a	0.42
Ca	0.93a	0.19	0.72a	0.05	0.83a	0.09	0.61a	0.06
Mg	0.65b	0.03	0.73ab	0.06	0.69ab	0.02	0.79a	0.03
Fe	0.056a	0.01	0.066a	0.01	0.039a	0.00	0.075a	0.02
Mn	0.119a	0.02	0.051b	0.01	0.136a	0.01	0.075b	0.01
Zn	0.059a	0.04	0.017a	0.00	0.016a	0.00	0.014a	0.00
Cu	0.009a	0.00	0.010a	0.00	0.010a	0.00	0.010a	0.00
Al	0.117a	0.06	0.060a	0.01	0.020a	0.00	0.027a	0.01
Na	0.620a	0.12	0.547ab	0.07	0.439ab	0.05	0.316b	0.04

Table S2 Acorn element:P ratios (molar) of *Q. variabilis* and *Q. acutissima* at P-deficient and P-rich sites in central Yunnan Plateau, Southwest China. Means with different letters differ significantly ($p < 0.05$) among the four groups of acorn species by site combinations.

Acorns	*Q. variabilis*				*Q. acutissima*			
	P-deficient sites		P-rich sites		P-deficient sites		P-rich sites	
	Mean	SE	Mean	SE	Mean	SE	Mean	SE
C:P	1155.94a	41.01	951.62b	47.54	1147.25a	61.23	848.78b	63.25
N:P	16.77a	1.08	13.06b	0.83	15.44ab	0.91	12.54b	0.74
S:P	0.68a	0.07	0.54a	0.06	0.65a	0.05	0.53a	0.04
K:P	6.07a	0.24	5.27a	0.34	6.17a	0.30	5.69a	0.32
Ca:P	0.75a	0.17	0.47ab	0.04	0.62ab	0.06	0.35b	0.04
Mg:P	0.85a	0.05	0.76a	0.04	0.85a	0.03	0.74a	0.04
Fe:P	0.033a	0.01	0.031a	0.01	0.021a	0.00	0.035a	0.01
Mn:P	0.067a	0.01	0.025b	0.01	0.075a	0.01	0.030b	0.00
Zn:P	0.031a	0.02	0.006a	0.00	0.007a	0.00	0.005a	0.00
Cu:P	0.004ab	0.00	0.004ab	0.00	0.005a	0.00	0.003b	0.00
Al:P	0.128a	0.06	0.058a	0.01	0.022a	0.00	0.025a	0.01
Na:P	0.809a	0.11	0.606a	0.07	0.567a	0.05	0.321b	0.05

Table S3 Weevil larva element concentrations (mg g^{-1}) in *Q. variabilis* and *Q. acutissima* at P-deficient and P-rich sites in central Yunnan Plateau, Southwest China. Means with different letters differ significantly (p < 0.05) among the four groups of acorn species by site combinations.

Weevil larvae	*Q. variabilis*				*Q. acutissima*			
	P-deficient sites		P-rich sites		P-deficient sites		P-rich sites	
	Mean	SE	Mean	SE	Mean	SE	Mean	SE
C	633.08a	12.69	639.57a	9.41	644.91a	1.52	635.72a	2.26
N	53.64a	1.37	55.88a	1.43	54.70a	0.59	57.08a	2.43
P	9.38b	0.18	10.03a	0.14	9.46b	0.18	10.39a	0.19
S	3.53a	0.19	3.51a	0.20	3.07a	0.24	3.58a	0.29
K	12.41b	0.20	12.50b	0.28	12.29b	0.29	13.79a	0.35
Ca	0.89b	0.07	1.00b	0.09	0.81b	0.02	1.36a	0.25
Mg	3.68c	0.11	4.12a	0.10	3.74bc	0.13	4.06ab	0.10
Fe	0.132b	0.02	0.340b	0.11	0.116b	0.02	0.646a	0.20
Mn	0.096a	0.01	0.076b	0.00	0.098a	0.01	0.094a	0.01
Zn	0.126a	0.01	0.118a	0.01	0.115a	0.00	0.140a	0.01
Cu	0.091a	0.05	0.055a	0.01	0.048a	0.00	0.055a	0.01
Al	0.101bc	0.03	0.195ab	0.05	0.056c	0.01	0.292a	0.09
Na	0.726b	0.31	1.253ab	0.59	0.223b	0.02	2.319a	0.78

Table S4 Weevil larva element:P ratios (molar) in *Q. variabilis* and *Q. acutissima* at P-deficient and P-rich sites in central Yunnan Plateau, Southwest China. Means with different letters differ significantly ($p < 0.05$) among the four groups of acorn species by site combinations.

Weevil larvae	*Q. variabilis*				*Q. acutissima*			
	P-deficient sites		P-rich sites		P-deficient sites		P-rich sites	
	Mean	SE	Mean	SE	Mean	SE	Mean	SE
C:P	175.21ab	4.04	166.27ab	3.49	176.68a	3.78	162.87b	2.35
N:P	12.76a	0.40	12.57a	0.35	12.84a	0.23	12.56a	0.63
S:P	0.36a	0.01	0.34a	0.02	0.31a	0.02	0.33a	0.03
K:P	1.05a	0.01	0.99b	0.02	1.03ab	0.01	1.05a	0.02
Ca:P	0.07b	0.00	0.08b	0.01	0.07b	0.00	0.10a	0.02
Mg:P	0.50a	0.01	0.52a	0.01	0.50a	0.01	0.50a	0.01
Fe:P	0.008b	0.00	0.018b	0.01	0.007b	0.00	0.034a	0.01
Mn:P	0.006a	0.00	0.004b	0.00	0.006a	0.00	0.005ab	0.00
Zn:P	0.006a	0.00	0.006a	0.00	0.006a	0.00	0.006a	0.00
Cu:P	0.005a	0.00	0.003a	0.00	0.002a	0.00	0.003a	0.00
Al:P	0.012bc	0.00	0.022ab	0.01	0.007c	0.00	0.032a	0.01
Na:P	0.102b	0.04	0.161ab	0.07	0.032b	0.00	0.297a	0.10

Table S5 Probability values of the effects of site type (P-deficient and P-rich sites) and specie (*Q. variabilis* and *Q. acutissima*) on element concentrations in acorns, weevil larvae and soils. Bold values are significant at $p < 0.05$.

Elements	Acorns			Weevil larvae			Soils		
	species	sites	species×sites	species	sites	species×sites	species	sites	species×sites
C	0.344	0.364	0.526	0.495	0.938	0.435	**0.003**	**0.000**	**0.022**
N	0.799	0.961	0.274	0.683	0.100	0.963	**0.002**	**0.000**	**0.002**
P	0.104	**0.000**	0.247	0.403	**0.000**	0.435	0.195	**0.000**	0.085
S	0.390	0.875	0.469	0.275	0.339	0.245	——	——	——
K	**0.001**	**0.003**	**0.036**	0.123	**0.012**	**0.015**	0.172	0.104	**0.020**
Ca	0.340	0.119	0.997	0.408	**0.007**	**0.048**	0.745	**0.000**	0.337
Mg	0.144	**0.023**	0.694	0.934	**0.001**	0.601	0.420	**0.000**	0.277
Fe	0.851	0.177	0.427	0.292	**0.000**	0.093	0.778	**0.000**	0.968
Mn	0.367	**0.000**	0.834	0.123	**0.030**	0.184	0.075	**0.000**	0.228
Zn	0.354	0.448	0.487	0.737	0.455	0.073	0.640	**0.011**	0.099
Cu	0.338	0.812	0.354	0.413	0.553	0.448	0.208	**0.000**	**0.037**
Al	0.079	0.526	0.421	0.907	**0.001**	0.121	0.934	**0.000**	0.762
Na	**0.022**	0.283	0.783	0.844	**0.009**	0.092	0.706	**0.003**	0.294

Table S6 Mean concentrations (mg g^{-1}) and coefficient of variation (CV%) of acorn and weevil larva elements in both *Q. variabilis* and *Q. acutissima* across all the variable nutrient sites in central Yunnan Plateau, Southwest China.

Elements	Acorns		Weevil larvae	
	Mean	CV%	Mean	CV%
C	443.42	5.04	639.06	4.70
N	7.40	18.96	54.99	7.94
P	1.14	20.93	9.75	7.66
S	0.70	25.72	3.41	24.55
K	8.25	15.63	12.65	8.92
Ca	0.79	51.73	0.98	43.93
Mg	0.71	17.19	3.88	11.72
Fe	0.06	83.31	0.27	141.43
Mn	0.10	56.37	0.09	24.81
Zn	0.03	298.05	0.12	27.65
Cu	0.01	24.20	0.06	158.98
Al	0.06	201.26	0.15	122.52
Na	0.49	58.64	1.01	175.16

Table S7 Geographic locations (latitude, LAT; longitude, LON; altitude, ALT) and conditions (height, SH; diameter at breast height, DBH; soil pH) of the selected *Quercus variabilis* and *Quercus acutissima* stands at P-rich (near Kunming) and P-deficient (near Chuxiong) sites in Yunnan, China.

Sites	Codes	LAT (°)	LON (°)	ALT (m)	SH (m)	DBH (cm)	Soil pH
Q. variabilis stands							
Fulongcun, Chuxiong	FL	25.24	101.53	1846	16.6	26.1	5.09
Zhongtunshuikun, Chuxiong	ZT	25.44	101.46	1849	15.7	33.2	5.46
Qingrengu, Chuxiong	QR	25.07	101.61	1862	19.8	28.4	5.13
Jiuba, Chuxiong	JB	25.06	101.58	1829	14.5	27.3	4.73
Heilongtan, Kunming	LT	25.14	102.75	1939	10.5	40.8	5.21
Zhiwuyuan, Kunming	ZW	25.14	102.74	1964	9.7	33.5	5.52
Sanjiacun, Kunming	SJ	24.85	102.59	1800	13.0	32.6	7.87
Wenquan, Kunming	WQ	24.98	102.44	1848	11.0	26.8	5.35
Q. acutissima stands							
Qingrengu, Chuxiong	QR	25.07	101.61	1862	14.1	30.4	5.13
Jiuba, Chuxiong	JB	25.06	101.58	1829	12.9	29.7	4.73
Makoushang, Chuxiong	MK	25.08	101.61	1814	11.3	31.8	4.78
Qiumuyuan, Kunming	QM	24.98	102.45	1937	11.3	31.6	5.03
Beitacun, Kunming	BT	24.94	102.46	1850	10.0	28.3	6.40
Jilecun, Kunming	JL	24.92	102.51	1851	9.5	24.3	5.89

Table S8 Chemical compositions of phosphate rocks (%) in Anning County (based on Tao [1]), one of P-rich sites in Kunming City, and non-phosphate rocks (the Matoushan Formation rocks) (%) in Mouding County (based on Shi, et al. [2]), one of P-deficient sites in Chuxiong City, in Yunnan Province. Measurement data that are not available are indicated by the symbol /.

	Non-phosphate rocks			Phosphate rocks		
	Mean	SD	n	Mean	SD	n
SiO_2	64.28	9.89	20	20.36	7.53	10
CaO	8.45	2.06	20	40.65	4.61	10
Al_2O_3	7.46	2.70	20	1.08	0.66	10
Fe_2O_3	1.45	1.04	20	1.33	0.66	10
MgO	2.32	1.56	20	1.39	1.46	10
P_2O_5	0.09	0.03	20	27.97	3.22	10
K_2O	1.93	0.77	20	/	/	/
FeO	1.38	0.69	20	/	/	/
MnO	0.09	0.02	20	/	/	/
Na_2O	0.81	0.43	20	/	/	/
BaO	0.04	0.01	20	/	/	/
Others	11.21			7.23		

Table S9 Detection limits and standards for all elements.

Elements	Detection limits	Detection standards
C	0.01%	Sulfanilamide (Elementar Art-No.:15.00-0062)
N	0.01%	Sulfanilamide (Elementar Art-No.:15.00-0062)
P	0.08 mg kg^{-1}	a multi-element standard solution (Custom Assurance Standard, Lot#:27-027CR, Cat#:XCCC-13A, SPEX CertiPrep®, USA),
S	0.05 mg kg^{-1}	a multi-element standard solution (Custom Assurance Standard, Lot#:34-002CR, Cat#:XCCC-14A, SPEX CertiPrep®, USA),
K	0.01 mg kg^{-1}	a multi-element standard solution (Custom Assurance Standard, Lot#:27-027CR, Cat#:XCCC-13A, SPEX CertiPrep®, USA),
Ca	0.01 mg kg^{-1}	a multi-element standard solution (Custom Assurance Standard, Lot#:27-027CR, Cat#:XCCC-13A, SPEX CertiPrep®, USA),
Mg	0.02 mg kg^{-1}	a multi-element standard solution (Custom Assurance Standard, Lot#:27-027CR, Cat#:XCCC-13A, SPEX CertiPrep®, USA),
Fe	0.01 mg kg^{-1}	a multi-element standard solution (Custom Assurance Standard, Lot#:27-027CR, Cat#:XCCC-13A, SPEX CertiPrep®, USA),
Mn	0.02 mg kg^{-1}	a multi-element standard solution (Custom Assurance Standard, Lot#:27-027CR, Cat#:XCCC-13A, SPEX CertiPrep®, USA),
Zn	0.02 mg kg^{-1}	a multi-element standard solution (Custom Assurance Standard, Lot#:27-027CR, Cat#:XCCC-13A, SPEX CertiPrep®, USA),
Cu	0.03 mg kg^{-1}	a multi-element standard solution (Custom Assurance Standard, Lot#:27-027CR, Cat#:XCCC-13A, SPEX CertiPrep®, USA),

Al	0.02 mg kg^{-1}	a multi-element standard solution (Custom Assurance Standard, SPEX CertiPrep®, USA),	Lot#:27-027CR, Cat#:XCCC-13A,
Na	0.01 mg kg^{-1}	a multi-element standard solution (Custom Assurance Standard, SPEX CertiPrep®, USA),	Lot#:27-027CR, Cat#:XCCC-13A,
